# Relationship between Dry Eye Disease and Dyslipidemia: A Systematic Review

**DOI:** 10.3390/jcm12206631

**Published:** 2023-10-20

**Authors:** Tzu-Hao Wang, Yuan-Jen Tsai, Yuan-Hung Wang, Chien-Liang Wu, I-Chan Lin

**Affiliations:** 1 Division of General Medicine, Department of Medical Education, Shuang Ho Hospital, New Taipei City 23561, Taiwan; 22265@s.tmu.edu.tw; 2 Graduate Institute of Biomedical Informatics, College of Medical Science and Technology, Taipei Medical University, Taipei City 11031, Taiwan; 3 Department of Family Medicine, Taipei Medical University Hospital, Taipei City 11031, Taiwan; 223025@h.tmu.edu.tw; 4 Graduate Institute of Clinical Medicine, College of Medicine, Taipei Medical University, Taipei City 11031, Taiwan; 12072@s.tmu.edu.tw; 5 Department of Medical Research, Shuang Ho Hospital, Taipei Medical University, New Taipei City 23561, Taiwan; 6 Department of Ophthalmology, School of Medicine, College of Medicine, Taipei Medical University, Taipei City 11031, Taiwan; eyedrwu@w.tmu.edu.tw; 7 Department of Ophthalmology, Wan Fang Hospital, Taipei Medical University, Taipei City 11696, Taiwan

**Keywords:** dry eye disease, dry eye syndrome, dry eye, dyslipidemia, hyperlipidemia, hypercholesterolemia, hypertriglyceridemia, systematic disease

## Abstract

Background: Dyslipidemia has been suggested to be associated with the occurrence of dry eye disease (DED). However, whether dyslipidemia is responsible for the development of DED remains unclear. In this systematic review, we explored the relationship between DED and dyslipidemia by using quantitative data. Methods: Following the Preferred Reporting Items for Systematic Reviews and Meta-Analyses guidelines, we conducted a comprehensive literature search in several databases, including PubMed, Embase, Cochrane Library, Web of Science, and Google Scholar, and obtained six relevant studies. Results: Our findings indicated that the majority of the selected studies reported a statistically significant association between dyslipidemia and DED, particularly in women. However, our quantitative analysis revealed that only two studies reported statistically significant differences in total cholesterol and high-density lipoprotein cholesterol values. Conclusion: No statistically significant differences exist in the majority of lipid profile parameters between individuals with and without DED, but there is a statistically significant association between dyslipidemia and DED.

## 1. Introduction

Dry eye disease (DED) is a chronic and multifactorial condition that progressively affects a significant number of individuals worldwide. This condition is also exacerbated by the prolonged and frequent use of visual display terminals and excessive occupational exposure to toxic agents, such as fire, smoke, dust, and airborne particles [1,2,3,4]. With a continuously increasing prevalence of approximately 7% to 33%, DED has been identified as a critical worldwide public health problem [5], placing a substantial burden on public health sectors [6,7].

**Figure 1:**
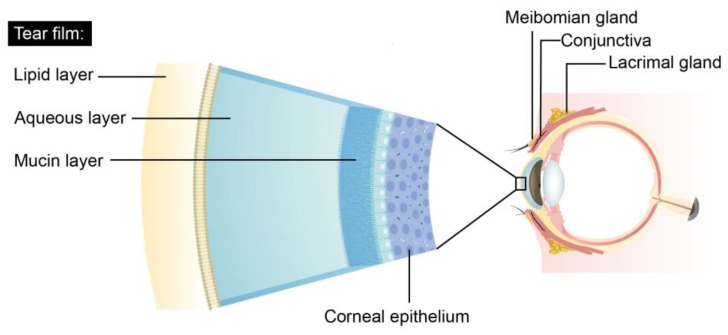
The three layers of the tear film.

The primary hallmark of DED is the disruption of the tear film, resulting in reduced aqueous tear flow and excessive evaporation [8]. As shown in Figure 1, the tear film consists of lipid, aqueous, and mucin layers [9,10]. The lipid layer plays a major role in maintaining a smooth corneal surface, preventing rapid evaporation from the eye [11], and protecting the eye against environmental stressors. This layer primarily contains secretions from the meibomian glands [12], with cholesterol and cholesterol esters serving as key components [13]. Because the meibomian glands play a crucial role in regulating lipid secretion within the eyelids, systemic dyslipidemia, characterized by increased levels of total cholesterol (TC), low-density lipoprotein cholesterol (LDL-C), and triglycerides (TGs) and decreased levels of high-density lipoprotein cholesterol (HDL-C), may influence the development of DED.

Many epidemiological studies have indicated a correlation between elevated cholesterol levels and meibomian gland dysfunction (MGD), a key factor in the pathogenesis of DED [14]. However, the independent relationship between dyslipidemia and DED still remains under debate and has not been rigorously studied. Given the increasing number of individuals affected by DED, ophthalmologists must investigate the association between dyslipidemia and DED to provide further insights. Because of the inconclusive nature of the relationship between dyslipidemia and DED [15,16,17], we conducted a systematic review to consolidate the existing data and clarify the existing debate. We hope that this systematic review will contribute to future clinical practices associated with the early diagnosis and treatment of DED and will provide valuable guidance in this field.

## 2. Materials and Methods

### 2.1. Search Strategy and Data Retrieval Protocol

We conducted a systematic literature review using various electronic databases, including PubMed, Embase, Cochrane Library, Web of Science, and Google Scholar, from their inception until March 2023. We used the following terms to search for publications on DED and dyslipidemia: “dyslipidemia” AND “dry eye”, “hyperlipidemia” AND “dry eye”, “cholesterol” AND “dry eye”. “hypercholesterolemia” AND “dry eye”, “triglyceride” AND “dry eye”, “hypertriglyceridemia” AND “dry eye”, “low-density lipoprotein” AND “dry eye”, and “high-density lipoprotein” AND “dry eye”. We then manually screened the reference lists of the selected articles to identify any potentially relevant publications that we may have missed.

**Figure 2:**
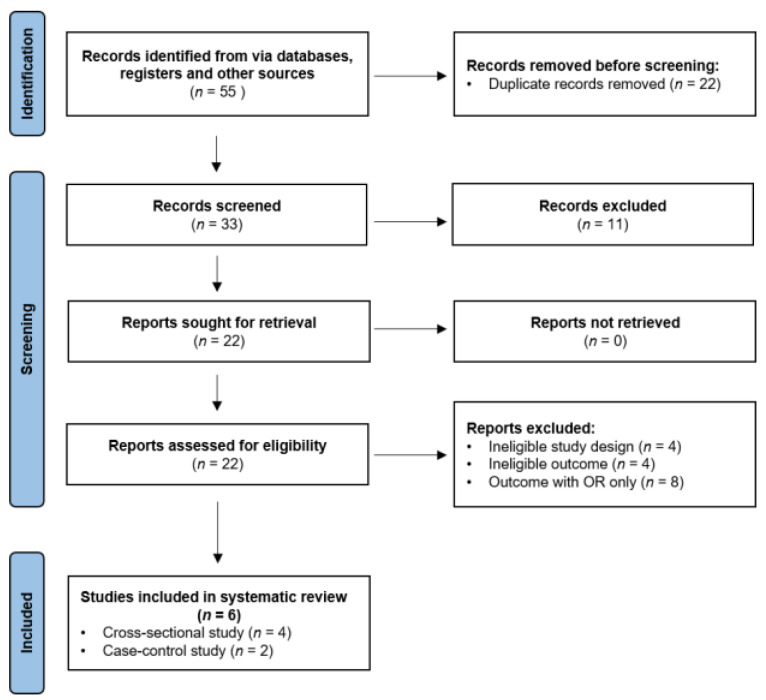
PRISMA flow diagram of the study selection process and evaluation outcomes.

In adherence to the Preferred Reporting Items for Systematic Reviews and Meta-Analyses (PRISMA) criteria [18] and the International Prospective Register of Systematic Reviews (PROSPERO) guidelines [19], a structured and systematic search strategy was employed. The study selection process and evaluation outcomes are illustrated using a PRISMA flow diagram in Figure 2.

The initial search yielded 55 articles, 22 duplicates of which were removed. The remaining 33 articles were reviewed in terms of their titles and abstracts. After 11 articles that did not meet the inclusion criteria were excluded, the remaining 22 articles underwent full-text evaluation. Finally, following an eligibility evaluation, 6 articles were included and thoroughly examined.

### 2.2. Inclusion and Exclusion Criteria

Studies meeting the following criteria were included in this systematic review: (1) studies published in English, (2) studies involving peer-reviewed original research, (3) studies involving human participants, and (4) studies involving quantitative information on lipid profile parameters in individuals with and without DED. By contrast, studies meeting the following criteria were excluded from this systematic review: (1) studies reporting ineligible results, (2) studies focusing exclusively on MGD, and (3) studies possessing characteristics that contradict the aforementioned inclusion criteria. For more details, please refer to Table 1.

## 3. Results

A total of six studies were included in this systematic review: four cross-sectional studies and two case–control studies. Table 2 provides a summary of the characteristics of the included studies.

### 3.1. Highlights of Each Study

In the following text, we discuss the results of each of the aforementioned studies in detail.

Chun et al. [20] conducted a cross-sectional study on 5627 adults aged over 19 years, including 531 with DED. The information of all medication use was collected. They observed no significant gender-related differences in lipid profiles between individuals with and without DED. However, they observed an increased prevalence of high TC and LDL-C levels in older women with DED, particularly in those aged above 65 years. Adjusted analysis revealed that these elevated TC and LDL-C levels were associated with an increased likelihood of DED. Interestingly, they observed that women with DED had a lower prevalence of low HDL-C compared with the controls. They also observed that the mean value of HDL-C was higher in patients with DED, although not significantly, than in the controls. Notably, the authors used a DED questionnaire survey to identify potential patients with DED.

Park et al. [21] examined the relationship between dry eye syndrome (DES) and metabolic syndrome among adults aged 19 years and above. They observed no significant gender-related differences in TG or HDL-C levels between individuals with and without DES. However, they observed a significant association between elevated TG levels and DES in women. After adjustment for confounding variables, they determined that, compared with their counterparts, women with elevated TGs were 1.13 times more likely to have DES. Notably, this study included a total of 15,294 individuals, of whom 2704 received a diagnosis of DES based on signs, symptoms, and comprehensive eye examinations involving a slit lamp. No records of the prescription medication use of the individuals were mentioned.

Rathnakumar et al. [22] reported statistically significant differences in all serum lipid profile parameters, except for VLDL-C, between individuals with and without DED. They observed that women exhibited significant differences in each parameter. In particular, they observed a statistically significant difference in TC levels (*p* < 0.001) among women with DED, with a mean value of 363 ± 19.05 mg/dL. Notably, this study included a total of 60 clinically diagnosed DED cases and age- and sex-matched healthy controls. They only excluded patients using antihypertensive drugs. DED was diagnosed using a questionnaire and clinical examinations, such as the tear breakup time (TBUT) test, Schirmer’s test, and slit-lamp biomicroscopy.

Choi et al. [23] reported a significantly increased prevalence of hypercholesterolemia among patients with DES (*p* < 0.008). This prevalence increased with the severity of DES (*p* < 0.002). Only HDL-C exhibited a significant difference between individuals with and without DES, with those with DES exhibiting increased mean levels of HDL-C. After adjustment for confounding variables, the authors observed that, compared with men without dyslipidemia, men with dyslipidemia were 1.40 times more likely to have DES. However, they observed no significant associations between dyslipidemia and DES in women, regardless of their menopausal status. Notably, this study included a total of 2272 individuals, of whom 1117 received a diagnosis of DES based on Ocular Surface Disease Index (OSDI) scores. They excluded 147 individuals who were taking lipid-lowering drugs.

Shokr et al. [25] investigated the presence of microvascular endothelial dysfunction as an indicator of early cardiovascular disease in patients with DED. They observed significant differences in TC and HDL-C levels between individuals with and without DED, with *p* values of 0.036 and 0.014, respectively. However, they observed no significant differences in the other lipid profile parameters. Notably, this study included a total of 50 individuals, half of whom received a diagnosis of DED based on criteria set by TFOS DEWS II. Patients with the positive diagnosis of dyslipidemia that required medical treatment were excluded from the study.

Choi et al. [26] examined a total of 475 individuals, of whom 230 had DES. Individuals using lipid-lowering medication were included in the study. They defined DES as an OSDI score greater than 13. They observed no statistically significant association between the prevalence of hypercholesterolemia and DES or its severity.

### 3.2. Quantitative Data Analysis

In our quantitative analysis, we collected lipid profile parameters from each of the aforementioned studies (see Table 3). Among these parameters, only three exhibited a statistically significant difference between individuals with and without DED. Shokr et al. [25] reported a significant difference in the mean values of TC and HDL-C, with *p* values of 0.036 and 0.014, respectively. Choi et al. [23] reported a significant difference in the mean value of HDL-C, with a *p* value of 0.050. However, none of the other studies reported a significant difference in the lipid profile parameters between individuals with and without DED.

## 4. Discussion

In this systematic review, the scope was expanded to emphasize the relationship between DED and systemic dyslipidemia. Our analysis demonstrated a statistically significant association between dyslipidemia and DED, with a prominence in women. We also extracted the quantitative lipid profile parameters from each included study. There were no statistically significant differences in the values of lipid profiles between DED and non-DED patients.

### 4.1. Higher Prevalence of Dyslipidemia in Female DED Patients

According to the results of the selected studies, dyslipidemia is statistically significantly associated with DED in both men and women. However, compared with men with DED, women with DED tend to exhibit a higher prevalence of dyslipidemia.

Rathnakumar et al. [22] reported that DED was more significantly associated with elevated TC levels in women (*p* < 0.001) than in men (*p* < 0.002). They reported significantly higher TG levels and altered lipoprotein profile parameters (TC, increased LDL-C, and decreased HDL-C) in women than in men, indicating the predominance of dyslipidemia in women. Park et al. [21] reported a significantly increased prevalence of elevated TG levels in women with DES. After adjustment for confounding variables, they only observed a significant association between DES and hypertriglyceridemia in women in final multivariate models (*p* < 0.05). Chun et al. [20] reported statistically significant differences in the prevalence of high TC and LDL-C between the control group and women with DED stratified according to age (20–65 and above 65 years of age), especially among those aged above 65. After adjustment for confounding variables, they observed that the only lipid profile parameter associated with an increased likelihood of DED was elevated TC levels in women. In addition, other studies by Ooi et al. [27] and Mussi et al. [28] documented higher occurrences of DED symptoms and serum cholesterol levels in women compared to men.

The phenomenon of a higher prevalence of dyslipidemia in women with DED compared to men was observed in the studies mentioned above. Notably, Chun et al. [20] demonstrated the association of DED and dyslipidemia, especially among women aged above 65. The increased prevalence of dyslipidemia in women with DED aged >65 years can be attributed to the hormonal changes in women above age 60 [29,30,31,32]. Hjortland et al. [33] documented an increase in cholesterol levels that correlates with menopause, indicating a potential causal role of menopause in altering lipid levels. Carr et al. [34] observed that menopause was associated with a shift in LDL particles towards smaller, denser, and more atherogenic particles, in addition to higher LDL-C levels. Matthews et al. [35] also reported decreases in both total HDL-C and HDL2 in postmenopausal women. The increased risk of dyslipidemia in postmenopausal-aged women may be associated with the increased risk of dyslipidemia in older women with DED.

Despite the evidence provided by the majority of studies, Choi et al. [23] reported different results. After fully adjusting for age, body mass index, hypertension, diabetes, occupation, smoking and drinking habits, physical activity, contact lens use, computer use, region, and calendar year of study, they observed an adjusted odds ratio (OR) of 1.40 (1.03–1.90) for DES in men with dyslipidemia and an adjusted OR of 1.10 for DES in women with dyslipidemia, indicating that men with dyslipidemia are at an increased risk of DES compared with women. A stratified analysis of menopausal status revealed that the multiple adjusted ORs for DES were insignificant in both premenopausal and postmenopausal women. Also, another study by Ahn et al. [36] found that interactions between sex and dyslipidemia status among the DED patients showed no significance.

These contradictory results may be attributed to differing methodologies. In contrast to other studies, Choi et al. [23] evaluated the severity of DES through a questionnaire with OSDI scores, unlike other studies that employed more basic questions regarding eye dryness and prior DES diagnoses. It also incorporated multiple statistical models with potential confounders on the basis of previous studies and a larger sample size (*n* = 2272), enhancing the robustness of their results. Nevertheless, additional studies are still warranted to validate these inconclusive results.

### 4.2. Abnormal Lipid Profiles Are Not Significantly Associated with DED Patients

According to the data collected and integrated from each study (see Table 3), the majority of studies [20,21,26] revealed no statistically significant differences in lipid profile parameters between individuals with and without DED, despite the current prevalence of a strong association between DED and dyslipidemia.

However, some studies have highlighted a different point of view. For instance, Shokr et al. [25] reported that patients with DED had significantly increased TC and decreased HDL-C levels, albeit at borderline, consistent with previous studies. By contrast, Choi et al. [23] discovered that patients with DED had significantly increased HDL-C levels. Chun et al. [20] reported that women with DED had higher HDL-C levels than those in the control group. They also indicated that, among women, none of the combinations of lipid profile parameters that included low HDL-C exhibited significant differences in prevalence between individuals with and without DED, whereas all combinations without low HDL-C exhibited significant differences. In summary, according to Chun et al. [20], Park et al. [21] and Choi et al. [23], patients with DED have elevated HDL-C levels, although the difference is not statistically significant, which contradicts the current perception of HDL-C as a well-known preventive factor in cardiovascular disease, indicating that HDL-C may have negative effects on the presence of DED.

The reasons underlying why elevated HDL-C levels may pose a risk for the development of DED, despite the established role of HDL-C in heart protection, remain unclear. Multiple studies have directly investigated this phenomenon by using relevant animal models. However, only a few studies have provided valuable insights, suggesting that the meibomian glands may be the key to unraveling this mystery [37,38]. The meibomian glands are a specialized type of sebaceous glands that are closely associated with the biosynthesis of cholesterol [39]. In a study involving a murine model, Deplewski et al. [40] discovered that both HDL-C and VLDL-C strongly promoted the differentiation of sebaceous epithelial cells, resulting in an increased accumulation of lipid droplets, which are the primary components of sebum. They also discovered that HDL-C induced the accumulation of both TGs and cholesterol. In another study involving a murine model, Yagyu et al. [41] discovered that the lack of acyl-coenzyme A:cholesterol acyltransferase-1 (ACAT-1) resulted in not only reduced atherosclerosis but also in the atrophy of the meibomian glands. ACAT-1 plays a crucial role in catalyzing the esterification of cellular cholesterol, facilitating the formation and release of lipoproteins into the bloodstream. Collectively, these studies indicate that, although HDL-C serves a cardioprotective function by facilitating the removal of endogenous cholesterol from tissues and transporting it to the liver for elimination, HDL-C may contribute to MGD through lipid production. However, further investigation of the precise biochemical mechanisms underlying the involvement of HDL-C in MGD is warranted to provide valuable insights.

### 4.3. Limitations

This review has several limitations. First, of the six studies reviewed, four had a cross-sectional design and two had a case–control design. These studies confirmed a link between dyslipidemia and DED, but were unable to establish a causal relationship. Therefore, well-designed longitudinal studies are warranted to further clarify the relationship between DED and lipid profile parameters. Secondly, Rathnakumar et al. [22], Choi et al. [26] and Shokr et al. [25] included small samples in their studies, meaning that the statistical power of their studies may have been insufficient to validate their findings and their results may not be representative of the general population. Although the remaining studies included larger samples, they all solely targeted the Korean population, resulting in a lack of genetic and ethnic variability and increasing the difficulty of reproducing the results to in more heterogenous populations.

Thirdly, no standardized diagnosis of DED has yet been established. All of the included studies utilized questionnaires, such as the OSDI, as an evaluation metric of DED, which may have resulted in misclassification and recall bias due to the patients’ subjective responses. Despite the presence of conventional methods such as corneal staining, TBUT test, Schirmer’s test, and tear osmolarity test, no consensus has yet been reached regarding the diagnosis of DED [42,43].

Fourthly, not all potentially crucial confounding factors could be thoroughly controlled for or excluded from evaluations. Not all historical medical records were included and the influence of medical comorbidities was difficult to verify. In addition, potential environmental confounders and other influential factors, like the extent of computer or electronic device use or occupational elements [4], were also overlooked.

## 5. Conclusions

Dyslipidemia may be associated with DED, particularly in women with DED, who exhibit a significantly higher prevalence of dyslipidemia than men do. According to the quantitative data of relating to lipid profile parameters in the majority of the included studies, no statistically significant differences exist between individuals with and without DED. These results suggest that an abnormal lipid profile may not be directly linked to the development of DED. Despite this, the observed correlation emphasizes that ophthalmologists must be vigilant of regarding potential DED manifestations in female dyslipidemia patients. Early detection and intervention may prove essential in preventing or slowing the progression of DED.

## Figures and Tables

**Table 1:** Inclusion and exclusion criteria.

Inclusion Criteria
Studies discussing the potential involvement of dyslipidemia in DED Studies with peer-reviewed retrospective (cross-sectional studies, case–control studies) and prospective designs Studies involving quantitative information on TC, TGs, LDL-C, and HDL-C in individuals with and without DED
Exclusion criteria
Studies in languages other than English Studies not involving human participants Clinical guidelines, consensus documents, reviews, and conference proceedings Studies focusing exclusively on MGD Studies reporting qualitative data only

DED, dry eye disease; TC, total cholesterol; TG, triglyceride; LDL-C, low-density lipoprotein cholesterol; HDL-C, high-density lipoprotein cholesterol; MGD, meibomian gland dysfunction.

**Table 2:** Summary of the included studies regarding the relationship between DED and dyslipidemia.

Title	Country	Study Design	Case	Control	Diagnosis Method	Key Findings	References
Total cholesterol and lipoprotein composition are associated with dry eye disease in Korean women	South Korea	Cross-sectional study	531 cases (116 men, 415 women; age >19 years)	5096 controls (2292 men, 2804 women; age >19 years)	DED questionnaire survey	No statistically significant gender-related differences were observed in the values of TC, LDL-C, HDL-C, and TGs between individuals with and without DED. The prevalence of abnormal TC was significantly higher in women with DED than in their male counterparts. The prevalence of abnormal LDL-C was significantly higher in women aged over 65 with DED than in their male counterparts. The prevalence of low HDL-C was significantly lower in women with DED than in the control group.	Chun et al. [20] (June 2013)
The Association between Symptoms of Dry Eye Syndrome and Metabolic Outcome in a General Population in Korea	South Korea	Cross-sectional study	2704 cases (744 men, 1960 women; age >19 years)	12,590 controls (5782 men, 6808 women; age >19 years)	Delphi consensus method by a panel of 17 dry-eye experts	No statistically significant gender-related differences were observed in the values of TGs and HDL-C between individuals with and without DES. The prevalence of elevated TG was significantly higher in women with DES than in their male counterparts.	Park et al. [21] (April 2016)
Prevalence of dry eye disease and its association with dyslipidemia	India	Case-control study	60 cases (23 men, 37 women; age: 25–70 years)	60 controls (matched age and sex)	Questionnaire and clinical examinations	Compared with individuals without DED, individuals with DED (both men and women) demonstrated a statistically significant association with dyslipidemia (TGs, TC, HDL-C, and LDL-C values). The prevalence of dyslipidemia was significantly higher in women than in men.	Rathnakumar et al. [22] (November 2017)
Association Between Dyslipidemia and Dry Eye Syndrome Among the Korean Middle-Aged Population	South Korea	Cross-sectional study	1117 cases (332 men, 785 women)	1155 controls (522 men, 633 women)	OSDI	The prevalence of hypercholesterolemia was significantly higher in individuals with DES than in those without. The values of HDL-C were significantly higher in individuals with DES than in those without. A significant independent relationship was observed between dyslipidemia and DES in men after adjustment. No significant association between DES and dyslipidemia was observed in either premenopausal or postmenopausal women after adjustment.	Choi et al. [23] (July 2019)
Dry eye disease is associated with retinal microvascular dysfunction and possible risk for cardiovascular disease	United Kingdom	Case–control study	25 cases (12 men, 15 women; age: 35–50 years)	25 controls (15 men, 10 women; age: 35–50 years)	TFOS DEWS II criteria [24]	Compared with the control group, patients with DED had significantly higher values of TC and lower values of HDL-C.	Shokr et al. [25] (January 2021)
Risk Factors Influencing the Occurrence and Severity of Symptomatic Dry Eye Syndrome: A Cross-sectional Study	South Korea	Cross-sectional study	230 cases (77 men, 153 women; age >40 years)	245 controls (107 men, 138 women; age >40 years)	OSDI	The prevalence of DES was significantly higher in women than in men. Hypercholesterolemia and TC were not associated with DES or correlated with the severity of DES.	Choi et al. [26] (January 2021)

DED, dry eye disease; TC, total cholesterol; LDL-C, low-density lipoprotein cholesterol; HDL-C, high-density lipoprotein cholesterol; TG, triglyceride; DES, dry eye syndrome; OSDI, Ocular Surface Disease Index.

**Table 3:** Comparison of lipid profile parameters between individuals with and without DED.

Reviewed Article	Mean TC	Mean LDL-C	Mean HDL-C	Mean TGs
Chun et al. [20]				
Men				
Case	184.5 ± 3.4	110.4 ± 3.3	51.5 ± 1.5	134 ± 9.7
Control	187.2 ± 1	112.3 ± 0.9	49.6 ± 0.3	157.4 ± 3.9
*p* value	0.6799	0.8224	0.6395	0.7210
Women				
Case	185.8 ± 2.2	113.4 ± 1.9	56.3 ± 0.7	106.2 ± 4
Control	186.7 ± 0.9	113.9 ± 0.7	56 ± 0.3	109.3 ± 2
*p* value	0.4302	0.5698	0.2197	0.1337
Park et al. [21]				
Men				
Case			49.12 ± 11.63	149.30 ± 107.5
Control			49.20 ± 11.98	154.90 ± 127.20
*p* value			0.850	0.252
Women				
Case			55.01 ± 12.86	116.5 ± 72.72
Control			54.98 ± 12.80	115.0 ± 82.33
*p* value			0.930	0.453
Rathnakumar et al. [22]				
Men				
Case	275 ± 16.58	152 ± 12.3	38 ± 6.16	243 ± 15.58
Control				
*p* value				
Women				
Case	363 ± 19.05	171 ± 13.07	29 ± 5.38	328 ± 18.1
Control				
*p* value				
Choi et al. [23]				
Case	193.5 ± 35.0	112.6 ± 30.8	55.5 ± 14.0	110 [82, 155] *
Control	192.9 ± 35.6	112.7 ± 31.5	54.3 ± 13.4	115 [82, 159] *
*p* value	0.698	0.946	0.050	0.397
Shokr et al. [25]				
Case	193.35 ± 6.65	121.81 ± 8.89	46.79 ± 4.22	89.38 ± 6.19
Control	171.69 ± 7.35	108.66 ± 8.89	61.1 ± 3.48	83.19 ± 6.19
*p* value	0.036	0.301	0.014	0.502
Choi et al. [26]				
Case	188.4 ± 33.0			
Control	186.3 ± 36.6			
*p* value	0.532			

DED, dry eye disease; TC, total cholesterol; LDL-C, low-density lipoprotein cholesterol; HDL-C, high-density lipoprotein cholesterol; TG, triglyceride. * Median [interquartile range].

## Data Availability

Not applicable.
